# Family & bystander experiences of emergency ambulance services care: a scoping review

**DOI:** 10.1186/s12873-023-00829-3

**Published:** 2023-06-14

**Authors:** Eillish Satchell, Melissa Carey, Bridget Dicker, Haydn Drake, Merryn Gott, Tess Moeke-Maxwell, Natalie Anderson

**Affiliations:** 1 Te Ārai Palliative & End of Life Research Group, School of Nursing University of Auckland , Private Bag 92019, Auckland, 1142 New Zealand; 2 Paramedicine Research Unit, Auckland University of Technology, Auckland, New Zealand; 3 St John, New Zealand (Hato Hone Aotearoa), Auckland, New Zealand; 4 Adult Emergency Department, Auckland City Hospital, Auckland Mail Centre, Private Bag 92024, Auckland, 1142 New Zealand

**Keywords:** Family, Bystander, Emergency ambulance services, Paramedicine patient-family-centred care

## Abstract

**Background:**

Emergency ambulance personnel respond to a variety of incidents in the community, including medical, trauma and obstetric emergencies. Family and bystanders present on scene may provide first aid, reassurance, background information or even act as proxy decision-makers. For most people, involvement in any event requiring an emergency ambulance response is a stressful and salient experience. The aim of this scoping review is to identify and synthesise all published, peer-reviewed research describing family and bystanders’ experiences of emergency ambulance care.

**Methods:**

This scoping review included peer-reviewed studies that reported on family or bystander experiences where emergency ambulance services responded. Five databases were searched in May 2022: Medline, CINAHL, Scopus, ProQuest Dissertation & Theses and PsycINFO. After de-duplication and title and abstract screening, 72 articles were reviewed in full by two authors for inclusion. Data analysis was completed using thematic synthesis.

**Results:**

Thirty-five articles reporting heterogeneous research designs were included in this review (Qualitative = 21, Quantitative = 2, Mixed methods = 10, Evidence synthesis = 2). Thematic synthesis developed five key themes characterising family member and bystander experiences. In an emergency event, family members and bystanders described chaotic and unreal scenes and emotional extremes of hope and hopelessness. Communication with emergency ambulance personnel played a key role in family member and bystander experience both during and after an emergency event. It is particularly important to family members that they are present during emergencies not just as witnesses but as partners in decision-making. In the event of a death, family and bystanders want access to psychological post-event support.

**Conclusion:**

By incorporating patient and family-centred care into practice emergency ambulance personnel can influence the experience of family members and bystanders during emergency ambulance responses. More research is needed to explore the needs of diverse populations, particularly regarding differences in cultural and family paradigms as current research reports the experiences of westernised nuclear family experiences.

**Supplementary Information:**

The online version contains supplementary material available at 10.1186/s12873-023-00829-3.

## Background

In an emergency in the community, family members, caregivers, colleagues, neighbours or unknown bystanders may be required to summon assistance, direct ambulances, provide first aid, reassure the patient, recall events, provide background information and even act as proxy decision-makers. Individuals involved are likely to perceive emergency events differently [1] and when ambulance personnel respond to an emergency, it can be unclear how those present at the scene fit into the patient’s social network. Whatever that relationship, any event requiring an emergency ambulance response is likely to be salient and stressful for those involved [2].

This century there has been increasing research interest in paramedics’ experiences of working with bystanders and families during emergencies [3, 4]. However, recent reviews highlight the limited research from the perspective of family and bystanders during these events [5, 6]. The provision of *patient- and family-centred care* is an established approach within healthcare settings that emphasises the importance of working in partnership with patients and families to improve healthcare experiences [7, 8]. Dees [9] notes this requires a mental shift from a focus on what is done *to* the patient, to a consideration of what can be done *for* the patient, family and caregivers. Differing cultural, spiritual, or religious beliefs may affect the needs of family members and bystanders, and can be particularly important in crisis or end-of-life contexts where emergency ambulance services respond. For healthcare practitioners to provide effective patient and family-centred care which is culturally-safe [10], these needs must be recognised on an individual basis [11]. However, research shows that emergency medical personnel receive little training or clinical guidance on how to provide culturally safe-care [12, 13]. Accordingly, in order for ambulance personnel to provide patient and family-centred care it is necessary to understand the needs and experiences of families and bystanders who seek emergency ambulance care.

## Aim and objectives

The overall aim of this scoping review is to identify and synthesise all published, peer-reviewed research describing family and bystanders’ experiences of emergency ambulance care.

Objectives were to:

- Describe family and bystander experiences of emergency ambulance
- Identify what family and bystanders value about emergency ambulance care
- Understand how patient and family-centred care is applied in the unique emergency ambulance context
- Identify opportunities for future research

## Method

A scoping review method was selected to map heterogenous research describing family and bystander experiences and identify gaps in the literature. The review method was guided by the Joanna Briggs Institute (JBI) guideline for scoping reviews [14], and the Preferred Reporting Items for Systematic reviews and Meta-Analyses extension for Scoping Reviews (PRISMA-ScR) [15]. No formal registration of the scoping review protocol was completed.

### Eligibility criteria

Study inclusion criteria focused on studies that reported the experience of family and bystanders in out-of-hospital incidents where emergency ambulance services respond. Anticipating a relatively small amount of published research in this area, all studies describing family or bystander experiences of emergency ambulance care associated with medical events were eligible. Peer-reviewed full-length articles of all research designs including reviews were included to reflect the broad nature of the research question. As most research specific to the emergency ambulance context has been undertaken in the past 50 years [16, 17], no specific date limits were applied. Non-empirical articles such as commentary pieces and articles not available in English were excluded.

### Information sources & search strategy

Developing a robust search strategy in this area was a complex undertaking due to established challenges with indexing of emergency ambulance care [18] and qualitative literature [19]. Searching of literature consisted of two stages. In stage one (conducted in April 2022), a preliminary literature search of MEDLINE and Cumulative Index to Nursing and Allied Health Literature (CINAHL) was completed by authors ES and NA to trial and develop a comprehensive search strategy. Studies found in search one were reviewed by the research team and a subject librarian to identify additional search terms and create a comprehensive search strategy for search two. Search two (conducted in May 2022) applied search terms (see Table 1) using Boolean search operators to the following databases: Medline; CINAHL; Scopus; and PsycINFO. A search of ProQuest Dissertations & Theses database was also undertaken in the hope this could help us to identify resulting peer-reviewed publications.

**Table 1:** Key search terms

Search Concept	Search Terms
Emergency Ambulance Services & Out-of-hospital setting	paramedic* OR ambulance* OR emergency medical service* OR EMS OR emergency medical technician OR EMT OR first responder OR pre-hospital practitioner OR pre hospital OR pre-hospital OR out of hospital OR out-of-hospital
Family & Bystanders	Famil* OR Caregiver* OR Significant Other OR Parent OR Spouse OR Next of Kin OR NOK OR Witness* OR Bystander* OR whanau
Family/Bystander Experience	Experience* OR perspective*

### Data extraction

All search results were exported into Endnote 20 [20] for removal of duplicates and eligibility screening. Initial title and abstract screening was undertaken by author ES. Where discrepancies arose, these were resolved through discussion with other members of the authorship team. The full text of remaining studies were then screened independently against eligibility criteria by authors ES and NA. Electronic database searching was supplemented through manual searching of the reference lists of included studies. No formal critical appraisal of study methodologies was undertaken as the researchers included all studies meeting eligibility criteria in this review to recognise the heterogenous nature of studies and small quantity of literature found in search.

### Data synthesis

Studies were interrogated for findings relevant to the review objectives and these extracted results were compiled in Microsoft Word and uploaded to NVivo [21] where analysis of data was undertaken following the three-stage process of thematic synthesis as outlined in Table 2 [22]. Quantitative findings were largely descriptive in nature and these findings were converted to or included within descriptive themes. Coding and initial descriptive thematic development was primarily undertaken by ES and NA, with remaining authors involved in development and refinement of analytical themes.

**Table 2:** Thematic synthesis

Stage of Thematic Synthesis	Description of actions
Stage One: *Line-by-Line Coding*	Line-by-line coding of results was completed in NVivo. This process resulted in a total of 153 initial codes
Stage Two: *Development of Descriptive Themes*	Codes created during stage one were then reviewed for similarities and differences and were organised into groups. New codes were created to represent the grouped codes creating our descriptive themes., resulting in the creation of 16 of descriptive themes
Stage Three: *Development of Analytical Themes*	Researchers applied descriptive themes to answer the initial review questions. Five overarching analytical themes were created as a result of this process

## Results

### Selection of sources of evidence

**Fig. 1:**
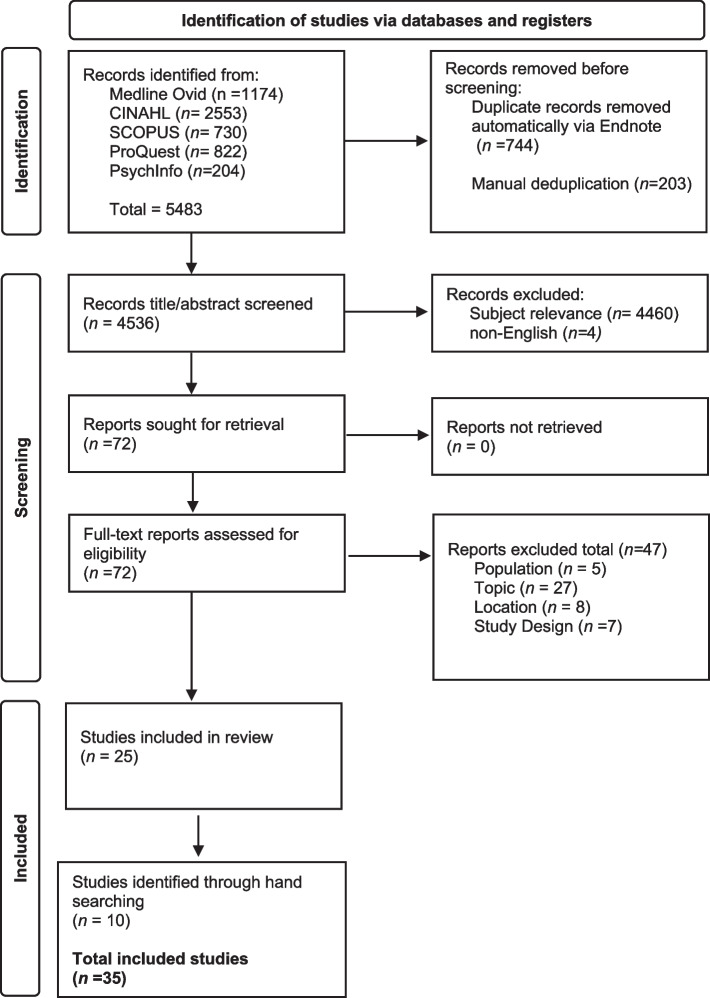
Prisma Flow Diagram. From: Page MJ, McKenzie JE, Bossuyt PM, Boutron I, Hoffmann TC, Mulrow CD, et al. The PRISMA 2020 statement: an updated guideline for reporting systematic reviews. BMJ 2021;372:n71. https://doi.org/10.1136/bmj.n71

As shown in Fig. 1 database searches and screening resulted in 25 articles selected for inclusion in this scoping review. All included article reference lists were hand-searched for additional studies resulting in the inclusion of a further 10 studies, bringing the included studies total to 35. Studies are summarised in Table 3.

**Table 3:** Included articles matrix

Author/Year/ Country	Aim/Objective (Verbatim)	Participants^a^	Research Design	Relevant Findings	Themes
Axelsson et al., (1996) [23] Sweden	To describe bystander-initiated CPR, the circumstances, & their experiences	742 bystanders who completed CPR between 1990–1994	Mixed Methods, phone interviews & postal questionnaires	93% of bystanders indicated that performing CPR was a mainly positive experience & reported positive interactions with ambulance crew. Bystanders had poor access to post-event debriefing opportunities	5
Axelsson et al., (1998) [24] Sweden	To identify the factors influencing the psychological reactions to performing CPR during an actual emergency reported by the bystanders	544 bystanders who completed CPR between 1992–1995	Mixed Methods, survey & telephone interviews	Two key factors influenced bystander psychological reactions: 1) Victim outcome, including unknown outcome; 2) Debriefing of bystanders post-event	5
Axelsson et al., (2000) [25] Sweden	To identify the factors influencing the psychological reactions to performing CPR during an actual emergency reported by the bystanders	19 bystanders who had performed CPR between 1997–1998	Qualitative, individual interviews	Five key themes were identified as affecting bystanders’ experience in performing CPR: 1: Acting out of humanity; 2: Competence of CPR; 3: Feelings of obligation; 4: Feelings of courage; 5: Feeling exposed	1, 2,
Bremer et al., (2009) [26] Sweden	To describe the experiences of significant others present at out-of-hospital cardiac events focusing on ethical aspects & values	Seven significant others	Qualitative, individual interviews	Significant others reported the following themes in their experience of out-of-hospital-cardiac arrest: unreality in reality; overwhelming responsibility; inadequacy & limitation; hope & hopelessness; ethical considerations; insecurity about the future; & the trembling of life	1, 2, 4
Carter et al., (2019) [27] Canada	To evaluate patient/family satisfaction & paramedic comfort & confidence in the provision of a novel palliative care in the home programme	18 family members & caregivers	Mixed methods, two-stage data collection (Part A patient/family & Part B paramedic) Mailed & online surveys, & phone interviews	The delivery of palliative care in the home by paramedics resulted in high patient/family satisfaction. Families & patients reported that receiving end-of-life care by paramedics in the home enabled care wishes to be fulfilled, peace of mind for the caregivers, & help during emergency situations	2, 4
Case et al., (2021) [28] Australia	To explore the psychological adjustment & experiential perspectives of survivors & families in the second year after out-of-hospital cardiac arrest (OHCA)	12 family members	Mixed methods, semi-structured interviews & a post-traumatic-stress-disorder (PTSD) psychological assessment	Family descriptions of persistent trauma symptoms & are at risk of developing psychological disorders such as PTSD, emphasising the distinct support needs of family post-event	2, 5
Critz. (1989) [29] USA	To describe the attitudes & experiences of family members with respect to death determination in the home for terminally ill patients	53 family members who experienced death determination of terminally ill patients in the home	Mixed methods, mailed survey consisting of set & free answer sections	There is a large variety of practices in terms of emergency medical services response to death determination of terminally ill patients in the home. Common feelings the family experience at this time are sadness, relief, & anger	2, 3, 5
Dainty et al., (2021) [30] Canada	Partnering with survivors & families to determine research priorities for adult out-of-hospital cardiac arrests	A diverse group with OHCA experiences including survivors, family members, health care professionals, & researchers	Mixed methods, survey & focus groups	Care required for families, bystanders & patients was identified as a top research priority. Post-event support was highlighted as important	5
De Stefano et al., (2016) [31] France	To understand family members’ experiences during CPR	30 family members who had family members die during an OHCA	Qualitative, phone interviews	Family presence during OHCA can help to ameliorate the pain of the death, through the feeling of having helped to support the patient during the passage from life to death & of having participated in this important moment	2, 3, 4, 5
Delbridge et al., (1996) [32] USA	To determine whether family members accept field termination of unsuccessful out-of-hospital cardiac arrest resuscitation	‘Closest Family member’ of 42 unsuccessful resuscitations terminated out-of-hospital (*n* = 25) & in ED (*n* = 17)	Quantitative prospective cohort study, structured interviews	Results suggest that family members accept termination of unsuccessful cardiac arrest resuscitations in the field & ED equally well. Furthermore, family members of patients for whom resuscitation efforts are terminated in the field, compared with those of patients who are first transported to an ED, show similar satisfaction with regard to the manner in which they were informed of the death & the overall care provided by the EMS system	3, 4, 5
Edwardsen et al., (2002) [33] USA	To determine the acceptance by family members regarding non-transport of patients in cardiac arrest following unsuccessful resuscitation occurring in private residences	33 adults from 21 families who were present at the time of the patient’s death	Mixed methods, Survey & telephone interviews	All 21 families (100%) of the non-transported patients were satisfied with both the medical care & the emotional support provided by EMS Family members of three of the 12 (25%) transported patients stated they would have preferred to have the patient die at home instead of being transported	3, 4
Fallat et al., (2019) [31] USA	To understand how family members view the ways EMS & other first responders interact with distressed family members during an intervention involving a recent or impending paediatric death	15 parents of seven deceased children or young adults who had interactions with first responders	Qualitative, parent Interviews	There was a minimal amount of awareness of family-centred practice by the various providers (EMS, police, ED staff & coroners) even in cases where the child was declared deceased on scene. Communication between providers & families was a key factor in the experience	3, 4, 5
Forsgarde et al., (2021) [34] Sweden	To describe extended collaboration in situations when an ambulance was called, as experienced by older patients, a significant other, & ambulance- & primary healthcare (PHC) centre personnel	An extended collaboration group of older patients (*n* = 3) & significant other (*n* = 1), ambulance personnel (*n* = 3) & PHC personnel (*n* = 4)	Qualitative, individual interviews using reflective lifeworld research (RLR) approach	Extended collaboration when an ambulance is called gives support in decisions through dialogue. Dialogue increases certainty through experience- & knowledge-sharing between all involved (patient, family, ambulance & PHC personnel)	2, 3
Forslund et al., (2008) [35] Sweden	To illuminate how spouses to persons with acute chest pain experienced the alarm situation, the emergency call & the prehospital emergency care	Nine significant others of adult patients requiring emergency medical services	Qualitative, individual interviews	Identified key feelings which spouses experience during a spouses acute chest pain. Themes included aloneness, uneasiness, & responsibility	1, 2
Holmberg et al., (2016) [3] Sweden	To elucidate meanings of the relationship with the clinicians in the emergency ambulance care setting as experienced by the patients’ significant other	Nine significant others of adult patients requiring emergency medical services	Qualitative, individual interviews	The main theme of ‘being lonely together’ was identified. Significant others share the struggle with the affected person while also experiencing loneliness as clinicians focus on the affected person. Actions of the clinicians affect SO feelings. SO feel acknowledged when listened to & comforted when care is provided to the patient	1, 2
Jabre et al., (2013) [36] France	The principal aim of this trial was to determine whether offering a relative the choice of observing CPR might reduce the likelihood of PTSD-related symptoms. We also assessed the effect of family presence on medical efforts at resuscitation & the well-being of the healthcare team	570 relatives of adult patients who were in cardiac arrest & were given CPR by 15 prehospital emergency medical service units	Randomised controlled trial	Family presence during CPR was associated with positive results on psychological variables & did not interfere with medical efforts, increase stress in the health care team, or result in medicolegal conflicts	4
Jarneid et al., (2020) [37] Norway	To investigate fathers’ experiences of being present at an unplanned birth outside a maternity facility	12 fathers	Qualitative, semi-structured interviews	Fathers’ experiences included stress, worry & anxiety but also pride & joy of stronger attachment to their partner & the baby they had helped to deliver. The support they received from the emergency services gave them increased reassurance & control, especially in cases where a midwife was present. Fathers sometimes did not feel confident in the care ambulance personnel could provide	1, 5
Jepsen et al., (2019) [38] Sweden	To explore the experiences of the caring encounter in the ambulance service among parents to children aged 0–14 years	14 parents of children aged 0–14 years old who were cared for by EMS	Qualitative, interviews	The parents described the importance of giving the family enough time in the situation, creating a safe environment & involving the parents in the care. In cases where the parents felt insecure, there had been lack of communication & lack of sensitivity, & the ambulance team did not invite the parents to participate in the care	1, 2
Jurhmann et al., (2022) [6] Australia	To review & synthesise the empirical evidence regarding paramedics delivering palliative & end-of-life care in community-based settings	23 articles. Articles which included ambulance staff (*n* = 20), patients (*n* = 4), family (*n* = 2)	Systematic integrative review	Paramedics can play an important role in facilitating home-based death & reducing avoidable hospital admissions. There is a strong desire amongst ambulance staff, family members & patients for paramedics to refocus their attention on holistic home-based management of palliative symptoms instead of hospital conveyance	1, 2, 4
Larsen et al., (2022) [39] Denmark	To explore relatives’ experience of out-of-hospital cardiac arrest during & post-event	12 relatives of adult OHCA survivors	Qualitative, semi-structured interviews	Relatives were challenged with witnessing OHCA & the trajectory after it, experiencing a high level of distress & anxiety. The development of support networks & education programs for patients & relatives is a critical element in supporting relatives of cardiac arrest survivors after discharge	1, 2, 5
Mathiesen et al., (2016) [40] Norway	To explore reactions & coping strategies in lay rescuers who have provided CPR to OHCA victims	20 lay rescuers who performed CPR	Qualitative, semi-structured interviews	Reactions after providing CPR to OHCA victims may cause serious & persistent concerns in lay rescuers. A common coping strategy was confiding in close relations. Some lay rescuers required professional help to cope with the OHCA incident	2, 5
Mausz et al., (2018) [41] Canada	To qualitatively explore bystander CPR to identify contextual influences on performance that might be relevant for CPR training & to describe the emotional & psychological impact of providing CPR	15 lay rescuers who were involved in an adult OHCA	Qualitative, semi-structured focus groups	Bystanders move through three key stages when encountering OHCA: being called to act; taking action & making sense of the experience. The long-term psychological consequences of bystander intervention in OHCA remain poorly understood & warrant further study	1, 2, 5
Merlevede et al., (2004) [42] Belgium	To determine the perceptions, needs & mourning reactions of their bereaved relatives confronted with sudden unexpected death & to assess the relationship with the cause of death	74 relatives of 53 deceased individuals who passed away suddenly out-of-hospital (*n* = 45) or in ED (*n* = 5)	Mixed methods, semi-structured interviews & standardised surveys	Common needs identified by the bereaved family were: lack of information/long waits for information; wanting an opportunity to view resuscitation or deceased; lack of family involvement in cases where the deceased is left in the home; & lack of follow up care	3, 4, 5
Moller et al., (2014) [43] Denmark	To explore the concept of debriefing bystanders after participating in an out-of-hospital cardiac arrest resuscitation attempt	15 bystanders who received telephone debriefing post-OHCA participation	Qualitative design, telephone interviews	Post-event debriefing when provided by healthcare professionals stimulates reflection, positively influencing the ability to cope with the emotional reactions & the cognitive perception of own performance; enhances motivation to perform CPR & motivates improvement of skills	4, 5
Myall et al., (2020) [5] United Kingdom	To identify the factors that shape & characterise experiences of prehospital practitioners, families & bystanders in the context of death & dying outside of the hospital environment where pre-hospital practitioners respond	51 papers included which reported on death & dying in the prehospital setting	Scoping review	Few papers focused on family & significant others’ experiences. Generally, relatives reported positive interactions with prehospital practitioners, & while some families reported more negative encounters, it suggests there may be a disconnect between prehospital practitioners’ perceptions of the care they provide & families’ experience of that care	1, 2, 4
Nordby & Nohr (2008) [44] Norway	To understand how relevant communicative challenges in cases of sudden infant death syndrome were perceived by both parents & paramedics	Six pairs of parents & six paramedics who had been involved in sudden unexpected death in infant	Qualitative, semi-structured interviews	Many of the parents interviewed were not satisfied with the paramedics' communication, empathy & ability to take care of the practical aspects of the situation	3, 4, 5
Nord-Ljungquist et al., (2020) [45] Sweden	To describe the emergency situation involving a while waiting for an ambulance assignment in a rural environment from the caller's perspective	Eight callers who alerted emergency services for another person in need of emergency help	Qualitative, individual interviews	Participants describe a double ambivalence between feeling alone in the situation & having full control, & trust handing over the responsibility yet losing control. Actions of emergency services can affect the experience of the caller	1, 2, 5
Peculo-Carrasco et al., (2020) [46] Spain	To determine the feelings of safety among patients & carers based on their experiences & those of their emergency care professionals	29 adult patients & 20 carers who requested emergency medical care, & 16 emergency care professionals	Qualitative, focus groups of patients, carers, & emergency professionals	The factors, elements or situations with the greatest influence on the perception of feeling safe in this study are related to information & communication, person-centred care & professional competency,	1, 2, 3
Peters et al., (2016) [47] Australia	To elucidate the experiences of family members after the loss of a loved one as a result of suicide	10 participants who were bereaved relatives of suicide	Qualitative, individual interviews	Interactions between those bereaved by suicide & first responders following a suicide can favourably or adversely influence the course of bereavement for loved ones. Participants identified that practical, emotional & financial support was needed to assist them with grieving & functional restoration	3, 5
Schmidt & Harrahill (1995) [48] USA	To better understand the perceptions, needs, & responses of family members after an out-of-hospital death	31 surviving family members of urban out-of-hospital deaths which were attended by paramedics	Mixed methods, surveys & interviews	Paramedics were found to be professional & supportive. All participants were satisfied with death determination in the home rather than transportation to a hospital. This pilot study suggests that paramedics are able to meet the needs of survivors at the time of an out-of-hospital death	3, 4, 5
Soontorn et al., (2020) [49] Thail&	To describe the experience of rural Thai family caregivers helping dependent elders during medical emergencies	15 family caregivers of elderly dependents experiencing medical emergencies	Qualitative, interviews	The setting of rural Thailand resulted in many challenges for family members in receiving emergency medical care. Barriers to emergency care were lack of home monitoring equipment, inexperienced caregivers in assessing warning signs, lack of information & understanding of emergency health services, & delayed arrival of EMS	2, 3
Swetenham et al., (2014) [50] Australia	To explore the introduction of an extended care paramedics rapid response palliative care team	24 carers of palliative care patients	Mixed methods, interviews with service users & surveys of paramedics	Extended care paramedics are able to meet the needs of patients & family members undergoing palliative care treatment	1, 3
Thoren et al., (2010) [51] Sweden	To describe spouses' experiences of witnessing their partners' cardiac arrest at home, including the time before the event & when it happened	15 spouses of deceased adult OHCA patients	Qualitative methodology, individual Interviews	Major domains were identified of ‘time before the cardiac arrest’ & ‘the cardiac arrest event’. Emergency call services are able to influence family members’ actions & experiences during these times	1, 2
Wisten et al., (2007) [52] Sweden	To elucidate the perceived support & the needs of bereaved parents confronted with sudden cardiac death of a child	Twenty-eight parents who experienced sudden cardiac death of a child	Qualitative, parent interviews	Positive factors were: perceived emotional support, being given time with the deceased, & a reconstruction of the circumstances at death by someone who could answer their questions. However, this study showed that a considerable proportion of the suddenly bereaved perceived a lack of support & information in the acute situation	1, 4, 5
Weslien et al., (2005) [53] Sweden	To provide insight into family members’ experiences of cardiac arrest	17 family members who witnessed cardiac arrest	Qualitative, semi-structured interviews	Three major phases occur for a family member witnessing cardiac arrest realisation of event, the arrival of EMS & takeover of care at the hospital. All of which impact the experiences of the family	1 – 5

^a^Only participants meeting eligibility criteria included in matrix table

### Study characteristics

Of the 35 included articles, 21 utilised a qualitive research design [25, 26, 31, 34, 35, 37–41, 43–47, 49, 51–55]; Two used quantitative design [32] (including one randomised control trial)[36]; two were review papers (one systematic integrative review[6], one scoping review)[5]; and 10 were mixed-methods [23, 24, 27–30, 33, 42, 48, 50]. The predominate population group of the included studies was family members with 18 studies solely focused on family perspectives [26, 29, 31–33, 35, 37–39, 42, 47–49, 51–55]. Some studies included family participants alongside other perspectives including patients [28] and emergency ambulance personnel [27, 36, 44]. Seven studies were classified as having a diverse participant population consisting of three or more different participant groups [5, 6, 30, 34, 45, 46, 50]. Perspectives covered across these studies were: family members, ambulance personnel, emergency services, bystanders, patients, primary health-care professionals, emergency department healthcare professionals, and health researchers. Six studies provided the perspective of lay rescuers/bystanders [23–25, 40, 41, 43].

The nature of the emergency event also varied between studies. Seventeen studies focused on out-of-hospital cardiac arrest [OHCA] [23–26, 28, 30, 32, 33, 36, 39–41, 43, 51–54]. A further five articles included a variety of incidents (including trauma, OHCA, suicide, and other medical emergencies) resulting in out-of-hospital death [5, 31, 42, 44, 48]. Four studies reported on emergencies in the context of palliative care [6, 27, 29, 50]. A single study reported on emergency ambulance care in the event of a suicide [47]. Lastly, eight studies described other medical emergencies where emergency ambulance treatment was given on scene prior to transportation of the patient to a healthcare facility [34, 35, 37, 38, 45, 46, 49, 55].

Studies included covered a date range of 1989 [29] till 2022 [6, 39]. A high proportion of studies came from the Nordic region (*n* = *17) *[23–26, 34, 35, 37–40, 43–45, 51–53, 55]. Other prominent study locations included North America (*n* = 8)[27, 29–33, 41, 48], Europe (*n* = 5) [5, 36, 42, 46, 54] and Australia (*n* = 4)[6, 28, 47, 50]. Only one study [49] took place in a non-westernised location (rural Thailand).

## Findings

Thematic Synthesis developed five overarching themes characterizing family and bystander experiences of emergency ambulance care. These are outlined in Table 4 and described below.

**Table 4:** Key themes

Theme	Description
*A world in chaos*	Recognising that a family member is having a medical emergency is a stressful and chaotic time for family members and bystanders
*Emotional extremes*	Family members and bystanders experience emotional extremes during an emergency medical event
*Communication is key*	Communication from emergency ambulance personnel is crucial in determining family members overall experience
*Family as partners*	Family members desire to have a meaningful presence during resuscitations and be involved in shared decision-making with ambulance personnel
*Fear of being left alone without answers*	The needs of family members and bystanders do not end when EMS leave. Low rates of support post-event are currently provided

### A world in chaos

Studies exploring the experience of family members [26, 37, 39, 52] and bystanders [25, 41] revealed feelings of chaos and confusion. A sense of panic often affected the ability of family members and bystanders to perform medical aid, with many describing an inability to think or act clearly [37, 41, 43, 45, 51, 53]. Bystanders and family members commonly reported that they did not have the knowledge to provide medical assistance while waiting for an ambulance to arrive [25, 26, 37, 51]. Wanting to help the victim but lacking the knowledge or ability to do so evoked a strong sense of powerlessness in family members and bystanders [37, 45, 51]. Powerlessness is demonstrated in an interview study exploring fathers’ experiences of being present at an unplanned out-of-hospital birth:

*“I’ve never felt at such a loss in my entire life (. . .) You’re sort of completely useless because you don’t know anything about this.” *[37]

Emergency ambulance arrival reduced family and bystanders’ feelings of chaos, bringing a sense of calm and control [5, 38, 45, 46, 50]. Family members reported a sense of relief to hand over the responsibility of care to paramedics [35, 38, 45, 53, 55]. *“…when the ambulance arrived, they took responsibility for the situation and I absolutely wanted them to do that … it felt good…”* [38]

### Emotional extremes

Throughout the event, bystanders and family members report emotional extremes, notably between hope and hopelessness [26] and chaos and calm [45]. Once they realised there was a medical emergency, family and bystander participants reported a sense of dread, and a fear that the person will die or has died [26, 28, 35, 39, 49, 54] Many family members and bystanders commenced basic life support in the hope that the victim could be saved [25, 41, 53]

*“I got it into my head that I would make him come back, I had that belief. I thought, I almost thought that I would make him come back.” *[25]

However, hopelessness grew amongst family and bystanders when they could not see any response to basic life support actions [25, 45]. This generated a sense of confusion among rescuers as to why their actions were unsuccessful and, in cases where a death occurred, caused a sense of guilt that their poor first aid may have affected the outcome [25, 26, 40, 41].

In the context of a life-threating emergency, calling for help from emergency ambulance services invoked a sense of hope that the victim could be saved [51]. Hearing approaching sirens in the distance or seeing ambulance personnel arrive on scene gave family and bystanders reassurance [45, 46, 53] Bystanders and families continued to hope that a ‘miracle could happen’ while ambulance personnel provided resuscitation to victims of cardiac arrests [26, 45]. However, as time progressed without the victim responding hope diminished and families were confronted with the possibility that bereavement may occur [26, 45, 53, 54].

*“because it was already 10 minutes that they’d been trying to resuscitate him, from when they arrived, at the end of 10 or 15 minutes, I asked if the heartbeat had resumed. They said no. It’s then that I started to understand that .. . well, that it was over!” *[54]

All hope on scene is lost when resuscitation efforts are terminated and death is confirmed [26]. In the case of transportation to a hospital, the pendulum of hope and hopelessness continues [26, 45].

### Communication is key

Communication was highlighted by family and bystanders to be a crucial factor in determining the overall experience with ambulance personnel [6, 44, 46, 54]. Clear communication with ambulance personnel made families feel informed and involved during emergencies [27, 54] The most frequent complaint regarding communication was a lack of information. Family were critical of poor communication from ambulance personnel, particularly in instances where there were several responders to the event. This was poignantly illustrated by a bereaved parent, who recalled *“I think there were seven persons in our house, but no one told me exactly what they were doing to our child.”*[44]. Families and bystanders often reported waiting for long periods for information [42, 53, 54]. Participants in several studies noted feeling frustrated that they couldn’t make sense of emergency personnel actions [38, 42, 44, 46, 54]. Excessive use of medical jargon left family members feeling excluded [38]. A key point of communication failure occurred when patients were transported to hospital. Watching the ambulance drive away with their loved ones was a time where family members expressed loneliness and helplessness [39, 44, 55], as demonstrated in this quote from a Swedish interview study of significant others experiences with ambulance care:

*“But I felt a little worried, because they put him on the stretcher and rolled him out, and then I had to ask; – What are you doing? Where is he going? We will take him to the ambulance and check the ECG, they said. Is he OK? … What shall I do? Shall I follow? You may decide for yourself, you can do whatever you want, they said. … But what shall I do? Shall I come back out? It was raining. … I knew was in good hands, but it was then I started to think: God, what is happening?” *[55]

When a death occurred, family members and bystanders report clearly remembering both comforting communication that conveyed kindness and compassion [5, 44, 47], and communication which lacked empathy or felt incentive [5, 47].

In the event of a death family were grateful when emergency services personnel took time post-resuscitation termination to stay on scene and provide psychological support [33, 48]. Helpful actions included notifying extended family [47], staying with family after the death until support had arrived [27, 47, 52], assisting with cares of the deceased [44, 50] as well as taking the time to talk with the family and answer any questions that they might have [33, 45, 48].

### Family as partners during resuscitation

While experiencing individualised care is an essential component of patient and family-centered care, no studies described how individual differences such as ethnicity, culture, spirituality, or religion affected the experience of family members or bystanders. Studies in the context of a cardiac arrest highlighted that families wanted the option to be present during resuscitation [36, 53, 54]. This was because some had started resuscitation themselves, wanted to support the patient (both physically and spiritually, and believed they would be helpful due to a knowledge of the patient’s medical history [54].

Common barriers family experienced to being present during resuscitation were: not being invited by ambulance personnel to be present [52, 53] or being asked to leave [44, 53]; a lack of room on scene [53]; concern that they might interfere with resuscitation [42]; and being afraid of what they might witness [54]. Several studies in this scoping review report on negative outcomes experienced by family who were excluded from resuscitation efforts [36, 42, 44, 54]. A large, randomised controlled trial concluded that family members who did not witness CPR were more likely to experience post-traumatic stress disorder symptoms 90 days post event [36].

Another important factor raised by families was that witnessing resuscitation efforts increased acceptance of outcome in the event of death [52–54]. In many of these instances, family reflected on the value of seeing the patient as deceased and witnessing ambulance personnel attempt everything possible to save the victim. This is demonstrated in the following extract from a randomised controlled trial where family members were given the option to witness out-of-hospital resuscitation efforts:

*“Having been a witness makes it possible to start to process the loss: ‘And I think that it's important, it's part of the work of grieving also, to see that everything was tried and to truly see it oneself, I think that's very important.” *[54]

Conversely, when family did not witness resuscitation efforts, they were left with questions: *“suddenly he passed away. But what happened the last 30 min? Even today, we don’t know exactly. That is still a gap in my memory that I need to fill in” *[53].

In the cardiac arrest context, families seemed accepting of termination of resuscitation on scene [32, 33, 48]. Some studies reported that family realised resuscitation efforts were not going to work earlier than the termination of resuscitation, and sometimes felt interventions went on for too long highlighting a lack of shared decision making between emergency ambulance personnel and families [26, 32, 48, 54].

*‘She [the ambulance nurse] was going on and on, and it was all lines [on the electrocardiogram monitor].’ So I thought, ‘Why do they carry on?’ That was my thought. ‘Why do they carry on for so long?’ I thought it would be better that they just pronounced [him dead].” *[26]

In the event of a death on scene, family members described that spending time with their loved was an important activity for psychological healing [42, 48, 52]. Viewing the deceased on scene gave family members time to say farewells, and supported family acceptance of the outcome [52]. One study highlighted that family members needed to be supported by ambulance personnel to view the deceased, as this was an emotional time for family [42].

### Post-event support: Fear of being left alone without answers

Participants in a number of studies reflected on the lack of psychological and practical support available to bystanders after emergency personnel depart the scene. Few studies reported formal debriefing or follow-up, so bystanders and family members frequently turned to friends and family for support [23, 24, 40, 43]. While support from friends and family was identified as important in psychological healing, many participants wished to speak to a healthcare professional about the event [24, 37, 40, 52]. Many family members and bystanders report that debriefing with healthcare professionals in instances where a death occurred, was important as it provided reassurance that their actions were sufficient [26, 43]. Having to make contact with support agencies was reported as a barrier by families with survivors stating that they found it difficult to reach out for help themselves [39, 43, 47].

Those present at the scene who were considered ‘bystanders’ rather than ‘family’ were less likely to receive any follow-up or information regarding the outcome of the event, usually due to privacy concerns [24, 40, 43]. Not knowing the outcome of the event was a significant source of distress for bystanders resulting in feelings of anger, worry and even guilt that they may not have done enough.

*“‘I wondered about the outcome. I looked in the newspaper. I checked to see if the flag was flying at half-mast. Did we manage this, or did we not?” *[40]

Conversely, learning about the outcome of the event—whether successful or not was strongly associated with a sense of closure [40, 41].

*“After hearing what happened, I finally felt at peace and I went home and I had a good night’s sleep for the first time in days and I just felt better. It’s a very sad situation, but I realize that I couldn’t have done anything different … I’ve got the answers. I’ve got the information I needed. I’ve had the rest, now it’s time to move forward.” *[41]

While participants in several studies reported emergency services stayed on scene for some time following termination of resuscitation [27, 47, 52] many still felt alone once ambulance personnel left [42, 44]. In the context of a death, family members felt in need of practical guidance [42, 53]. and some noted they were poorly informed when police and coronal investigations were required [29, 52].

## Discussion

This scoping review is the first to present a synthesis of research exploring family and bystander experiences of emergency ambulance care. We have identified five key themes which influence family and bystander experience of emergency events: a world in chaos; emotional extremes; communication is key; family as partners during resuscitation; and post-event support fear of being left alone without answers. Participants in all included studies experienced emotional extremes when witnessing an emergency. Communication with ambulance personnel was incredibly important in influencing the overall experience of family and bystanders. Where ambulance personnel provided effective communication, family and bystanders felt and informed and included. However, where communication was reported to be poor, family felt confused and isolated. Effective communication is well-established as a vital component of patient and family-centred care [56].

A key finding from this review was that those present at the scene of an emergency sometimes feel they are excluded and treated as unwanted or passive witnesses. Family and bystanders really valued ambulance personnel actions which acknowledged and engaged them and supported their presence and understanding. Emergency ambulance personnel have identified a number of barriers to family presence including a focus on medical treatment, concern resuscitation efforts would be compromised, safety on scene, and concern resuscitation would upset family members [5, 57, 58]. However, a recent systematic review highlights the established advantages and limited risks associated with supported family presence during resuscitation [59].

Providing culturally-safe care to family and bystanders is a crucial component of individualising patient and family-centred care [10, 60]. However, in this scoping review, we found that only three studies [36, 49, 54] incorporated any consideration into the varying needs of family/bystanders depending on their cultural or spiritual preferences. In many instances, demographic data which may aid researchers in determining the experiences of family members and bystanders was not collected.

Finally, the role of ambulance personnel does not end when a death occurs, as family and bystanders have demonstrated significant psychological needs during this time. Family valued the time and care given to them and their loved one by ambulance personnel post-resuscitation termination. However, many ambulance personnel feel unprepared to support family and bystanders in the event of a death, and need further access to training/resources to feel confident providing psychological support to the bereaved [61, 62].

### Family or bystander?

A challenge when developing this review was clearly defining the population of interest. It was often unclear how those present at the scene of a medical emergency were identified as ‘family member’. Furthermore, the term ‘bystander’ was used to describe participants who were strangers, acquaintances and family members who played a role in responding to the emergency whilst awaiting ambulance arrival. Typically, family member perspectives were sought from ‘next of kin’ based on a westernised heteronormative concept of a nuclear family (parent, spouse, or child). This potentially limits important perspectives of wider extended family and the experiences of significant others who are not direct kin. Future research is needed, which recognises that family structure can vary among different cultural paradigms [63].

## Limitations

A wide variety of terms are used to index research set in the prehospital context [64]. Despite researchers implementing a two-step search strategy and consulting with a subject librarian, a further 10 studies were identified through hand searching and reference searching, which potentially indicates that the database search strategy had low sensitivity.

## Conclusion

This scoping review has explored the experiences of family members and bystanders in the event of an emergency ambulance response. By incorporating elements of patient and family-centred care into emergency practice, ambulance personnel can have a significant impact on the overall experience of family members and bystanders. In comparison to literature exploring paramedic perspectives there is limited research exploring the experiences of family and bystanders in the pre-hospital setting. Most of the existing research focuses on the contexts of cardiac arrest, but this is only a fraction of emergency ambulance work. Exploration of family and caregiver experiences in other ambulance contexts including chronic disease management, mental health care and non-conveyance situations, is also warranted. There is a distinct lack of literature exploring any family or bystander groups who may have differing ethnic, religious, or cultural backgrounds. More research – developed in consultation with service-users—is needed to identify the cultural and spiritual needs of family and bystanders when emergency ambulance services respond to ensure that care is provided is culturally safe.

## Supplementary Information

**Additional file 1:** **Supplementary File 1.** CINAHL Search Strategy.

## Data Availability

All data generated or analysed during this study are included in this published article [and its supplementary information files].
